# Supplementary material for: A loop region of BAFF controls B cell survival and regulates recognition by different inhibitors
Source: Nat Commun. 2018 Mar 23;9:1199. doi: 10.1038/s41467-018-03323-8 (PMC5865128; doi:10.1038/s41467-018-03323-8)
Supplement: Supplementary file 1 — Supplementary Information [file 41467_2018_3323_MOESM1_ESM.pdf]

SUPPLEMENTARY INFORMATION

A loop region of BAFF controls B cell survival and regulates recognition by different inhibitors.

Vigolo et al.

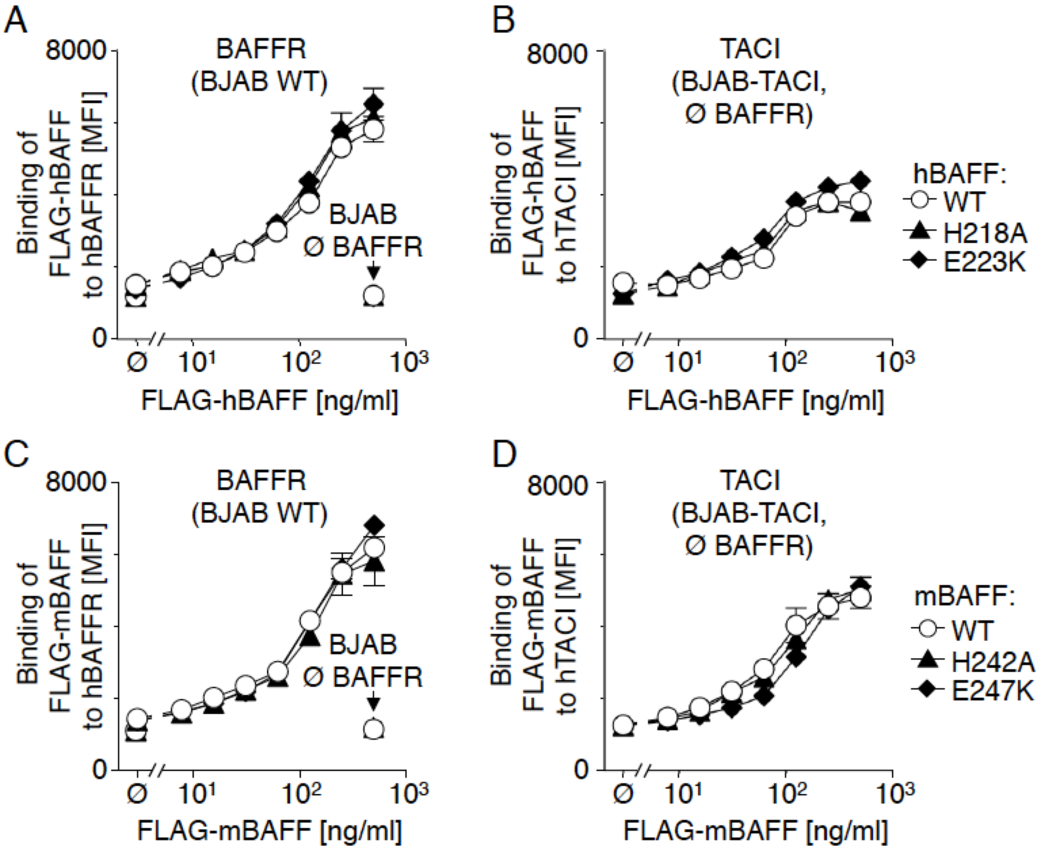

Supplementary Figure 1 (related to Figure 1). Mutations in the flap do not affect binding of BAFF to BAFFR and TACI on BJAB cells.

A. Titrated amounts of Flag-tagged human BAFF 3-mers (WT or with flap mutations) were added to BJAB cells, and binding was revealed with an anti-FLAG antibody. Binding of BAFF to BJAB BAFFR-CRISPR/Cas9-ko (Ø BAFFR) cells was also measured at the highest BAFF concentration.

B. Same as panel A, using BJAB Ø BAFFR cells expressing human TACI.

C. Same as panel A, but titrating FLAG-tagged mouse BAFF 3-mers.

D. Same as panel B, but titrating FLAG-tagged mouse BAFF 3-mers.

For panels A-D, each point shows the mean ± SEM of technical triplicates. Experiments of panels A-D were performed twice. MFI: mean fluorescence intensity.

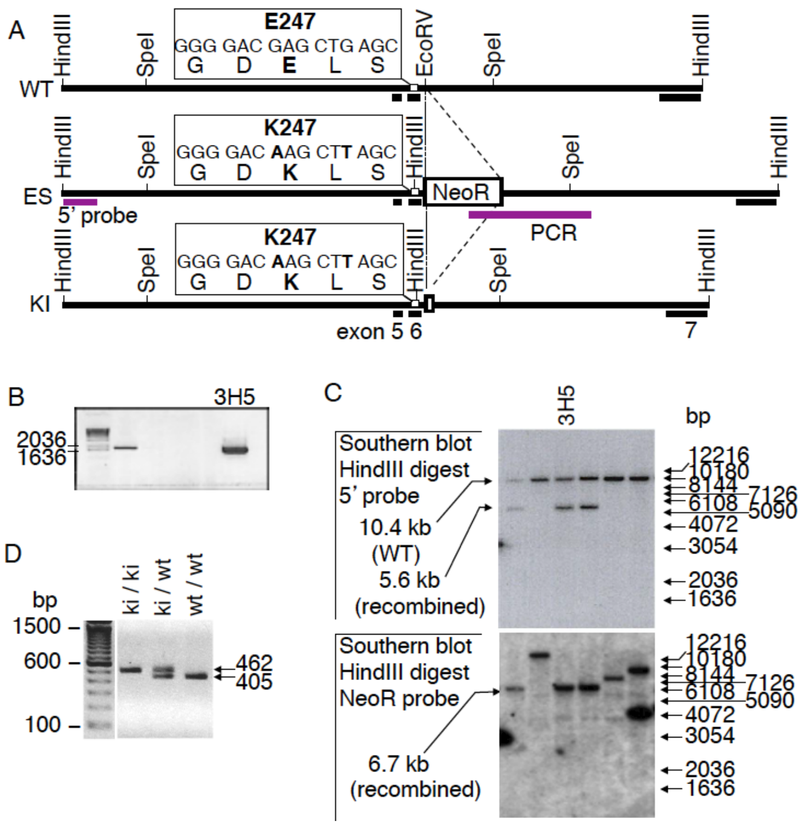

Supplementary Figure 2 (related to Figure 3). Generation of *Baff E247K* knock-in mice.

A. Schematic representation of the relevant sequence of the mouse *Baff* gene in wild type genome (WT), in recombined knock-in embryonic stem cells (ES) and in knock-in mice (KI) after deletion of the neomycin resistance (NeoR) cassette. Location of Baff exons 5, 6 and 7 are indicated as bold black lines. Positions of the PCR fragment amplified for screening recombined ES cells (see panel B) and of the 5' probe for southern blot (of panel C) are indicated as purple lines. Restriction sites of interest are also shown.

B. Identification of recombined clones of ES cells, including clone 3H5 that was selected for generating knock-in mice. Size markers (in base pairs) are shown on the left. This experiment was performed once.

C. Southern blot of HindIII-digested ES cell genomic DNA revealed with a 5' probe (top) and with a NeoR probe (bottom). This experiment was performed once.

D. Example of genotyping result for knock-in (ki/ki), heterozygous (ki/wt) and wild type (wt/wt) mice. Experiment performed once in this format (> 5 times in similar formats).

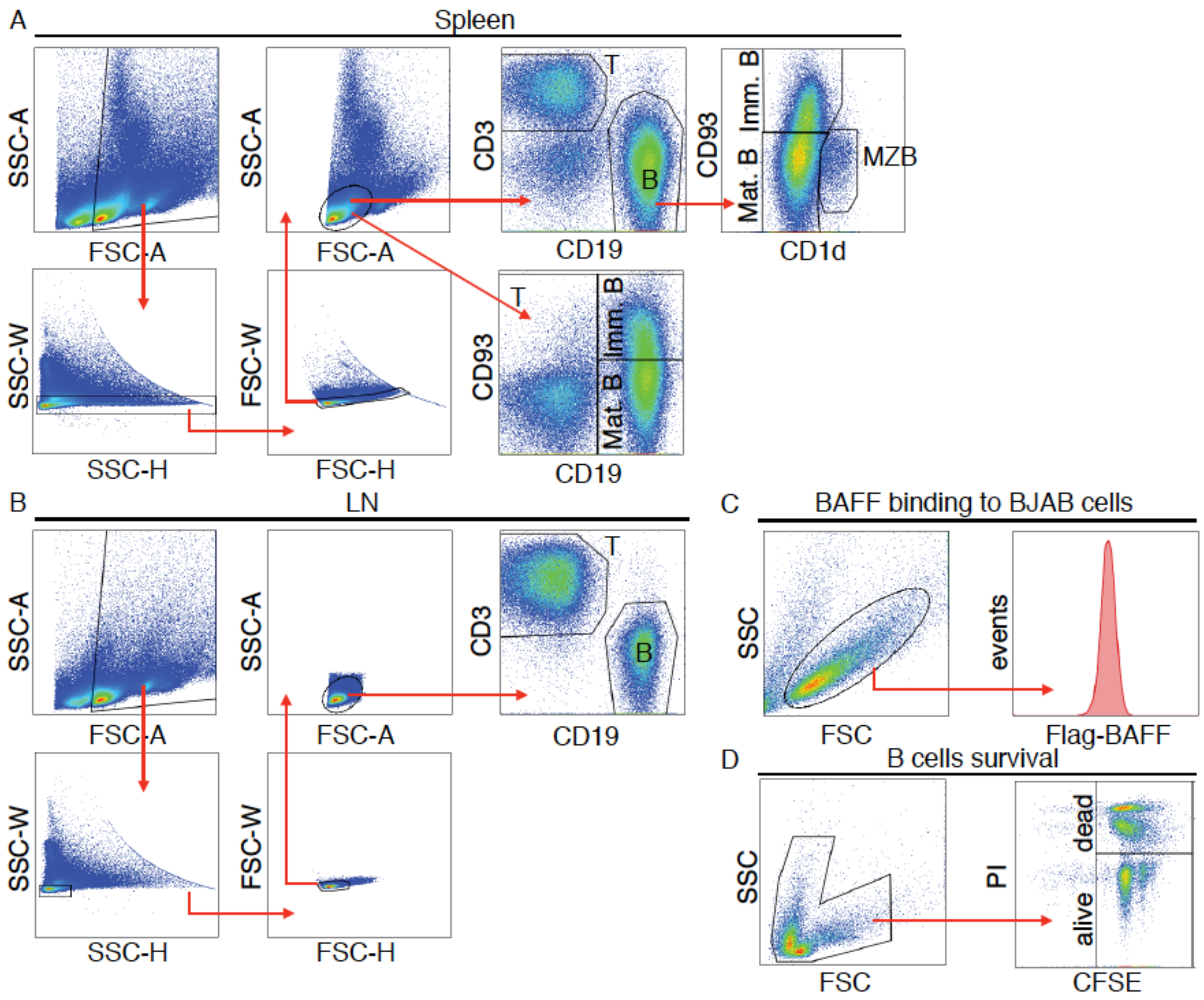

Supplemental Figure 3 (related to figures 3, 4, 5 and supplementary figure 1). Gating strategy for the identification of B cell subsets.

A. Gating strategy to identify B cells subsets in the spleen, for data shown in Fig. 3C-H, Fig. 5 and supplementary tables 1-3. SSC: side scatter. FSC: forward scatter. -W: width. -H: height. -A: area.

B. Same as panel A, but for lymph node cells.

C. Gating strategy used for supplementary figure 1.

D. Gating strategy for Fig. 4F. CFSE: carboxyfluorescein succinimidyl ester.

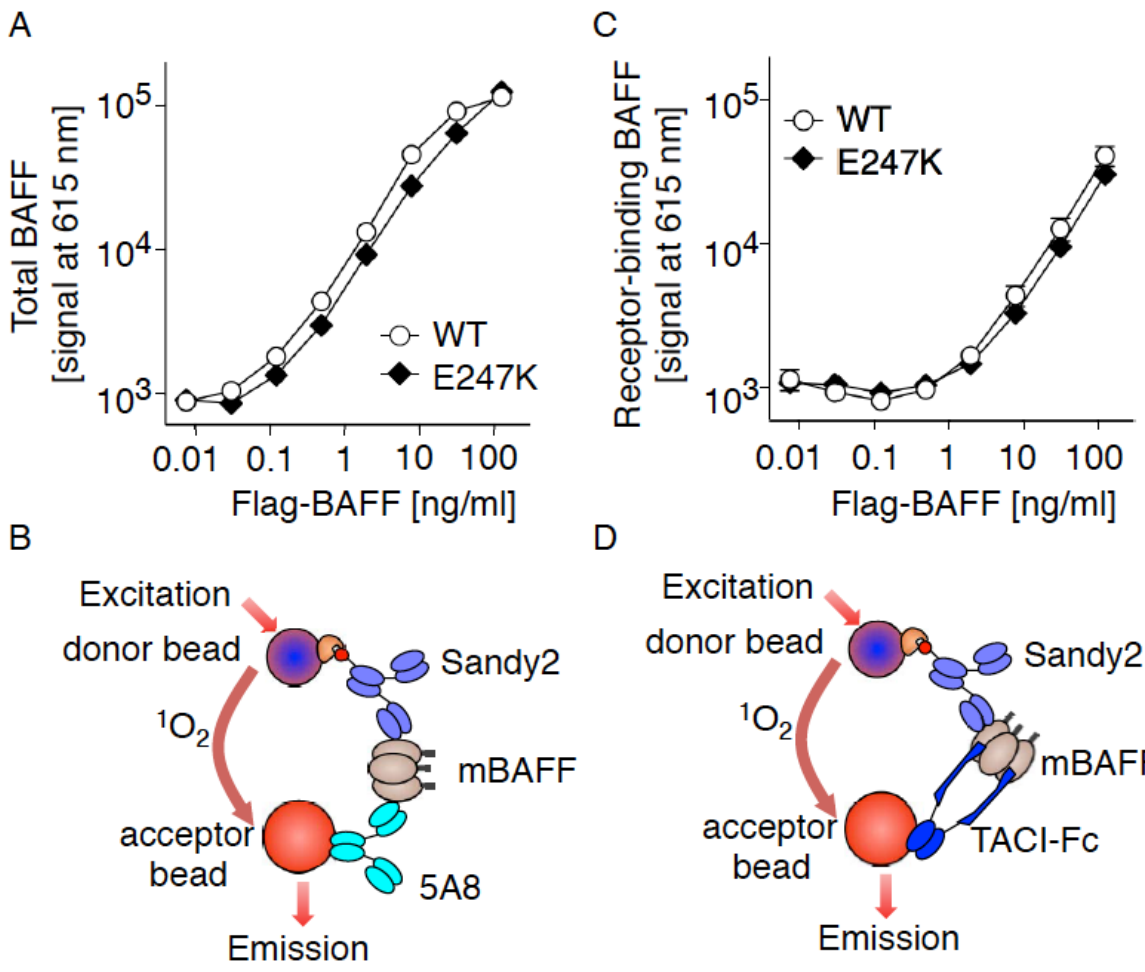

Supplementary Figure 4 (related to Figure 3). Detection of recombinant FLAG-mBAFF by AlphaLISA.

A. Detection by AlphaLISA of total FLAG-mBAFF (WT or E247K) at the indicated concentrations using 5A8-coupled acceptor beads and biotinylated Sandy-2.

B. Schematic representation of the assay used in panel A.

C. Detection by AlphaLISA of receptor binding-competent FLAG-mBAFF (WT or E247K) at the indicated concentrations using TACI-Fc-coupled acceptor beads and biotinylated Sandy-2. This experiment was performed twice.

D. Schematic representation of the assay used in panel C.

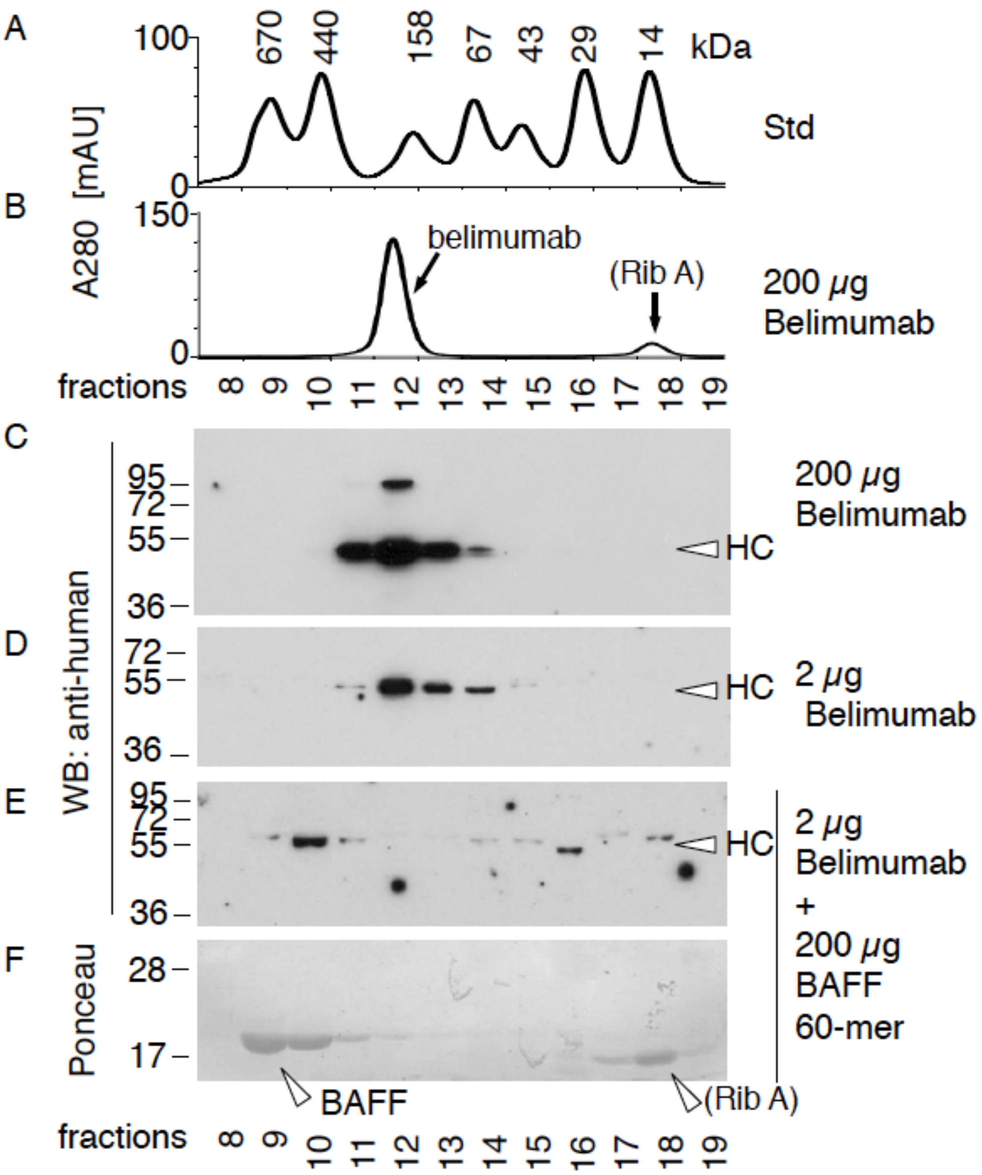

Supplementary Figure 5 (related to Figure 6). Belimumab does not bind to BAFF 60-mer.
Belimumab was incubated for 3 days with a 100-fold mass excess of BAFF 60-mer. The mixture was then size-fractionated by gel filtration chromatography. Belimumab was detected by Western blot in the eluted fractions, while the more abundant BAFF 60-mer was visualized by Ponceau staining of the membrane. Ribonuclease A (Rib A) was added in samples as an internal standard just before injection on the column.
A. Elution profile of molecular weight standards measured by absorbance at 280 nm. mAU: milli absorbance units.
B, C. 200 µg of un-complexed belimumab was detected by its absorbance at 280 nm, or by western blotting of eluted fractions. HC: heavy chain.
D. 2 µg of un-complexed belimumab was detected by western blotting of eluted fractions.
E, F. Elution profile of 2 µg of belimumab incubated with 200 µg of BAFF 60-mer. Belimumab was detected by western blotting (E) and BAFF 60-mer by Ponceau staining. Note that BAFF was 60-mer during the gel filtration, but dissociated into its 18.4 kDa monomeric constituents during the SDS-PAGE procedure. This experiment was performed once.

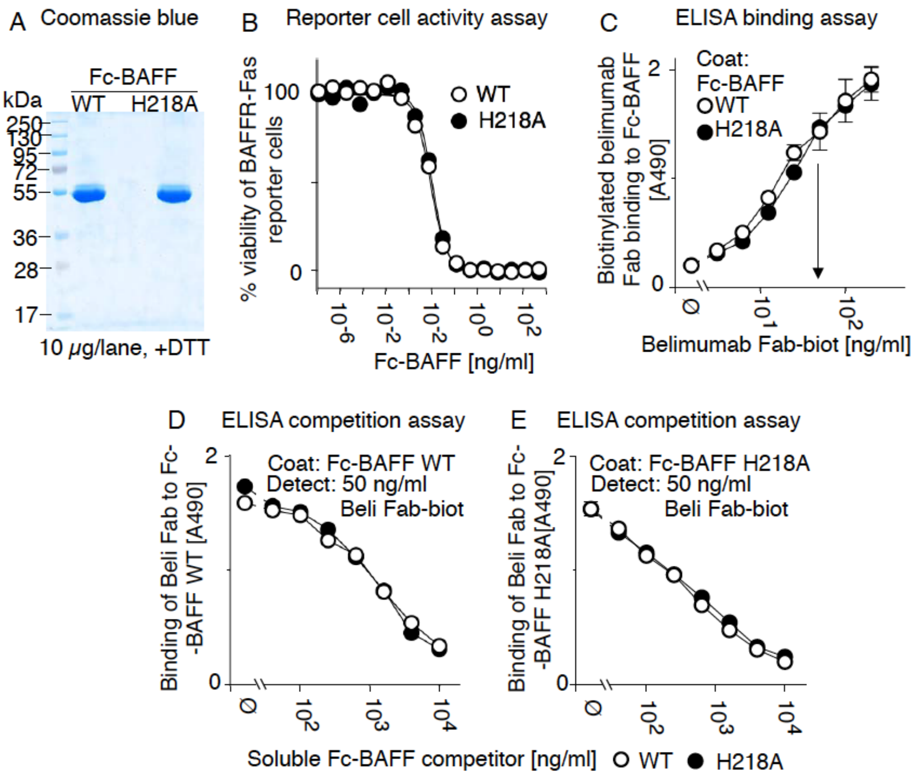

Supplementary Figure 6 (related to Figure 8). Mutation H218A in the flap of BAFF does not grossly alter the affinity for belimumab.

The fusion proteins Fc-BAFF, with or without the point mutation H218A in the flap of BAFF, were expressed in HEK 293T cells by transient transfection, then affinity purified on immobilized TACI-Fc. Fusion of a Fc moiety at the N-terminus of BAFF has at least two consequences: i) BAFF cannot form 60-mers for steric hindrance reasons and thus is recognized by belimumab (see Fig. 6A) and ii) fusion of a dimeric Fc to a trimeric ligand leads to the formation of active hexamers with two trimeric ligands intrinsically cross-linked by three dimeric Fc [1]. Fc-BAFF should thus be active regardless of mutations in the flap, and the flap should be accessible to belimumab.

A.- Coomassie blue staining of 10 µg of Fc-BAFF WT and Fc-BAFF H218A produced by transient transfection in HEK 293T cells and affinity purification on immobilized TACI-Fc.

B.- Fc-BAFF WT and H218A were incubated at the indicated concentrations for 16 h with BAFFR:Fas reporter cells, after which time cell viability was measured with the PMS/MTS cell viability assay. The experiment was performed twice.

C.- Fc-BAFF WT or Fc-BAFF H218A were coated in an ELISA plate. The biotinylated Fab fragment of belimumab was added at various dilutions, and binding to the coated Fc-ligand was detected with peroxidase-coupled streptavidin. The arrow indicates the concentration chosen for subsequent competition assays (see panels D and E). Mean ± SEM of duplicates.

D.- Fc-BAFF WT was coated in an ELISA plate. After the blocking step, soluble Fc-BAFF WT or H218A was added in wells at twice the indicated final concentrations. Without wash, biotinylated belimumab Fab was subsequently added and mixed immediately to reach a fixed final concentration of 50 ng/ml. After incubation and a washing step, bound Fab was detected as described in panel C. Mean ± SEM of duplicates.

E.- Same as panel D, except that Fc-BAFF H218A was coated in the ELISA plate. Mean ± SEM of duplicates. The experiments in panels C-E were performed twice.

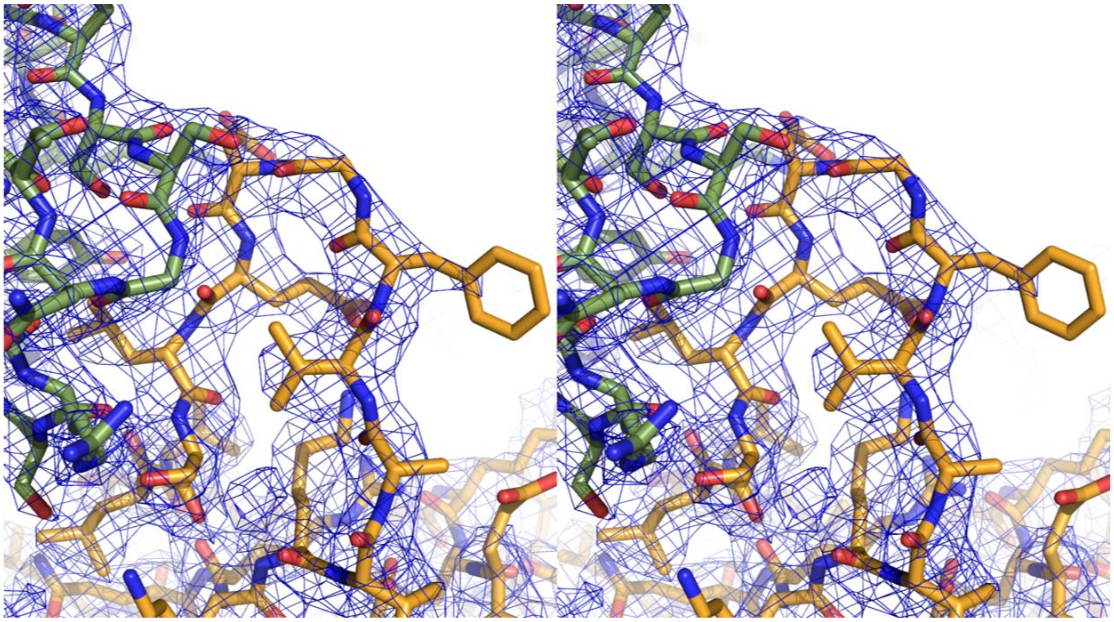

Supplementary Figure 7 (related to Figure 8). Stereo image of a portion of the electron density map of the BAFF – belimumab complex.

Stereo image showing a portion of the BAFF (yellow) - belimumab (green) complex around the flap loop of BAFF. The 2Fo-Fc electron density map contoured at 1 σ is shown as a blue mesh.

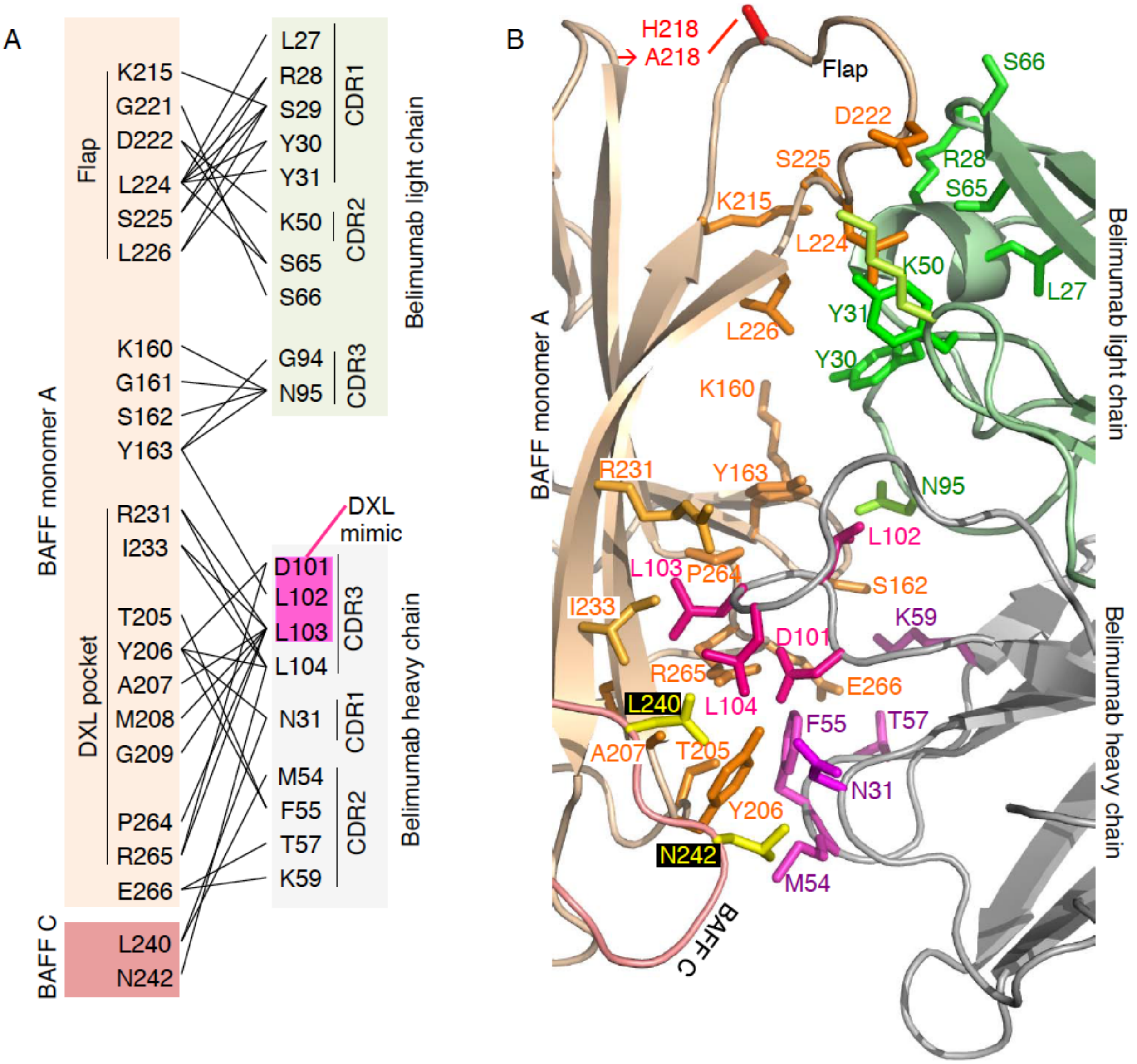

Supplementary Figure 8 (related to Figure 8). Interface of belimumab bound to BAFF.
A. Residues at the interface of the interaction between BAFF and belimumab that are closer than 4 Å are connected by black lines. CDR: complementarity-determining regions. DXL: Asp-Xxx-Leu motif. The DXL motif is at the centre of the binding of BAFF with its receptors (Fig. 8B, E).
B. Detailed view of the interaction site between belimumab and BAFF. Most residues at the contact site are identified. Mutation H218A (His218 → A218) that prevents assembly of BAFF 3-mer into 60-mers is also shown (but is not part of the BAFF-belimumab contact site).

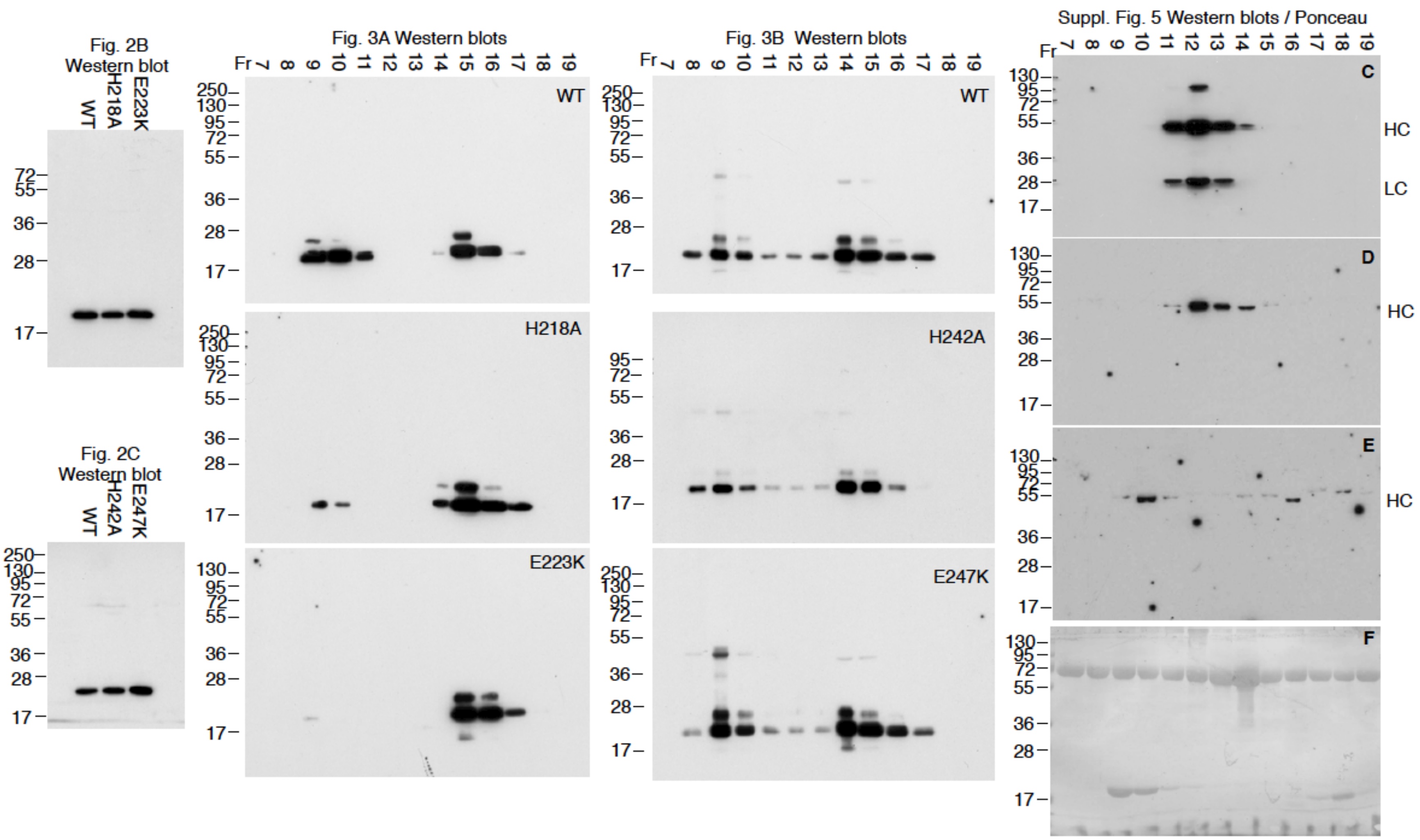

Supplementary Figure 9 (related to Fig. 2B, 2C, 3A, 3B and supplementary Fig. 5C, D, E, F). Uncropped WB (and Ponceau) images.
HC: heavy chain. LC: Light chain.

Supplementary Table 1 (related to Figure 3E, F). Lymphocytes counts in BAFF E247K knock-in mice and wild-type littermates compared to BAFF-deficient mice.

	Cell number (millions)				Cell frequency (%)			
BAFF genotype	+ / +	E247K / +	E247K / E247K	− / −	+ / +	E247K / +	E247K / E247K	− / −
n [a]	4	3	3	3				
Spleen								
All cells	42.9 ± 3.4 [b]	32.9 ± 4.0	29.0 ± 1.4	16.3 ± 1.1	100	100	100	100
T cells [c]	11.3 ± 0.7	10.5 ± 2.0	11.9 ± 1.8	9.22 ± 0.02	26.7 ± 2.1	31.2 ± 2.2	40.5 ± 4.2	57.2 ± 3.7
B cells [d]	25.7 ± 2.2	17.4 ± 2.1	8.7 ± 1.1	3.8 ± 0.7	59.8 ± 1.3	53.0 ± 2.2	29.7 ± 2.3	23.1 ± 2.3
Immature [e]	4.3 ± 0.3	4.5 ± 0.3	4.5 ± 1.1	2.1 ± 0.6	10.4 ± 1.4	14.0 ± 1.2	15.2 ± 3.0	12.4 ± 1.5
Mature [f]	20.3 ± 2.2	12.6 ± 1.9	4.2 ± 0.1	1.7 ± 0.1	46.9 ± 2.2	38.1 ± 2.6	14.5 ± 0.8	10.4 ± 0.7
MZ [g]	0.93 ± 0.20	0.32 ± 0.09	0.053 ± 0.008	0.048 ± 0.005	2.1 ± 0.3	0.96 ± 0.18	0.18 ± 0.03	0.29 ± 0.01
Lymph nodes [h]								
All cells	ND	ND	ND	ND	100	100	100	100
T cells	ND	ND	ND	ND	76.2 ± 1.9	83.6 ± 0.5	95.2 ± 0.4	96.9 ± 0.2
B cells	ND	ND	ND	ND	21.8 ± 1.6	14.6 ± 0.7	3.9 ± 0.4	1.2 ± 0.2
					Ratio			
B/T ratio					0.29 ± 0.03	0.18 ± 0.01	0.031 ± 0.005	0.013 ± 0.003

a) 8 to 12 weeks-old, randomized gender. b) mean ± SEM. c) $CD3^{+}$, $CD19^{-}$. d) $CD19^{+}$, $CD3^{-}$. e) $CD19^{+}$, $CD93^{+}$, $CD1d^{low}$. f) $CD19^{+}$, $CD93^{-}$, $CD1d^{-}$. g) $CD19^{+}$, $CD93^{-}$, $CD1d^{high}$. h) pooled inguinal, axillary and brachial lymph nodes.

Supplementary Table 2 (related to Figure 3G, H). Lymphocytes counts in BAFF E247K mutant heterozygous mice compared to wild-type heterozygous littermates.

	Cell number (millions)				Cell frequency (%)			
BAFF genotype	+ / +	+ / −	E247K / +	E247K / −	+ / +	+ / −	E247K / +	E247K / −
n [a]	3	5	4	4				
Spleen								
All cells	68.40 ± 2.84	33.71 ± 3.53	39.01 ± 6.65	24.97 ± 1.15	100	100	100	100
T cells [c]	29.23 ± 2.83	18.57 ± 1.73	24.79 ± 5.46	19.77 ± 0.83	42.57 ± 2.53	55.80 ± 2.78	61.95 ± 3.18	79.30 ± 1.32
B cells [d]	36.58 ± 1.41	12.14 ± 1.86	12.01 ± 1.13	3.54 ± 0.37	53.7 ± 3.16	35.02 ± 2.90	32.08 ± 2.73	14.10 ± 1.12
Immature [e]	3.78 ± 0.97	2.30 ± 0.61	1.91 ± 0.64	1.66 ± 0.29	5.55 ± 1.40	6.44 ± 1.29	4.47 ± 085	6.55 ± 0.97
T1 [f]	2.36 ± 0.73	1.60 ± 0.47	1.31 ± 0.50	1.33 ± 0.25	3.46 ± 1.05	4.50 ± 1.02	2.98 ± 0.74	5.22 ± 0.84
T2 [g]	1.34 ± 0.22	0.63 ± 0.14	0.56 ± 0.14	0.31 ± 0.04	1.96 ± 0.32	1.77 ± 0.27	1.39 ± 0.15	1.23 ± 0.11
Mature [h]	31.33 ± 0.75	9.50 ± 1.40	9.73 ± 0.47	1.87 ± 0.11	46.02 ± 2.68	27.61 ± 2.39	26.63 ± 3.39	7.28 ± 0.44
MZ [i]	0.528 ± 0.105	0.119 ± 0.024	0.136 ± 0.033	0.013 ± 0.001	0.765 ± 0.125	0.337 ± 0.060	0.358 ± 0.108	0.051 ± 0.005
Lymph node [j]								
All cells	7.92 ± 0.91	6.00 ± 0.37	5.60 ± 0.73	6.68 ± 1.07	100	100	100	100
T cells	5.89 ± 0.91	5.14 ± 0.33	4.70 ± 0.65	6.30 ± 0.97	73.70 ± 2.76	85.74 ± 1.77	83.60 ± 1.71	94.55 ± 1.05
B cells	1.71 ± 0.07	0.56 ± 0.12	0.58 ± 0.04	0.09 ± 0.02	22.20 ± 2.82	9.26 ± 1.82	10.81 ± 1.47	1.29 ± 0.19
					Ratio			
B/T ratio					0.305 ± 0.048	0.110 ± 0.024	0.130 ± 0.019	0.014 ± 0.002

a) 8 to 12 weeks-old, randomized gender. b) mean ± SEM. c) $CD3^{+}CD19^{-}$. d) $CD19^{+}CD3^{-}$. e) $CD19^{+}CD93^{+}CD1d^{low}$. f) $CD19^{+}CD93^{+}CD1d^{low}IgM^{high}IgD^{low}$. g) $CD19^{+}CD93^{+}CD1d^{low}IgM^{low}IgD^{high}$. h) $CD19^{+}CD93^{-}CD1d^{-}$. i) $CD19^{+}CD93^{-}CD1d^{high}$. j) pooled inguinal, axillary and brachial lymph nodes.

Supplementary Table 3 (related to Figure 5). Lymphocytes in BAFF E247K mice treated with anti-BAFF crosslinking antibodies compared to wild-type and BAFF$^{-/-}$ mice.

	Cell number (millions)					Cell frequency (%)				
BAFF genotype	E247K / E247K			− / −	+ / +	E247K / E247K			− / −	+ / +
Treatment	EctoD1 (ctrl)	Sandy5	5A8	−	−	EctoD1 (ctrl)	Sandy5	5A8	−	−
n [a]	6	6	6	5	6					
Spleen										
All cells	13.82 ± 1.73	22.13 ± 4.12	22.99 ± 2.39	21.03 ± 1.63	33.01 ± 4.90	100	100	100	100	100
T cells [c]	10.35 ± 1.39	12.33 ± 2.09	12.14 ± 1.16	14.97 ± 1.27	15.04 ± 2.27	74.58 ± 1.71	57.17 ± 2.72	53.12 ± 0.97	71.22 ± 3.77	45.88 ± 2.82
B cells [d]	2.11 ± 0.39	8.30 ± 1.96	9.33 ± 1.17	1.46 ± 0.10	14.49 ± 1.93	15.22 ± 1.72	35.52 ± 3.11	40.17 ± 1.63	6.99 ± 0.16	44.70 ± 1.65
Immature [e]	1.42 ± 0.29	2.79 ± 0.66	2.82 ± 0.51	0.95 ± 0.07	3.69 ± 0.33	10.33 ± 1.59	11.98 ± 1.16	11.90 ± 1.36	4.54 ± 0.23	11.98 ± 1.41
T1 [f]	0.62 ± 0.13	0.99 ± 0.26	0.92 ± 0.16	0.53 ± 0.04	1.10 ± 0.09	4.59 ± 0.85	4.12 ± 0.67	3.85 ± 0.40	2.52 ± 0.12	3.60 ± 0.52
T2 [g]	0.70 ± 0.15	1.56 ± 0.35	1.74 ± 0.33	0.33 ± 0.03	2.28 ± 0.23	4.98 ± 0.65	6.83 ± 0.33	7.31 ± 0.90	1.58 ± 0.09	7.36 ± 0.79
Mature [h]	0.65 ± 0.11	5.32 ± 1.26	6.26 ± 0.75	0.48 ± 0.04	10.06 ± 1.64	4.64 ± 0.36	22.72 ± 1.94	27.23 ± 1.44	2.27 ± 0.12	30.49 ± 1.53
MZ [i]	0.005 ± 0.001	0.092 ± 0.025	0.127 ± 0.023	0.012 ± 0.002	0.521 ± 0.086	0.038 ± 0.005	0.381 ± 0.052	0.545 ± 0.076	0.058 ± 0.004	1.552 ± 0.084
LN [j]										
All cells	ND	ND	ND	ND	ND	100	100	100	100	100
T cells	ND	ND	ND	ND	ND	94.38 ± 0.98	87.33 ± 0.35	83.13 ± 1.23	96.04 ± 0.77	77.73 ± 3.19
B cells	ND	ND	ND	ND	ND	3.67 ± 1.68	10.85 ± 0.35	15.35 ± 1.16	1.12 ± 0.13	20.22 ± 2.88
						Ratio				
B/T ratio						0.039 ± 0.008	0.124 ± 0.004	0.186 ± 0.018	0.012 ± 0.001	0.271 ± 0.053

a) 8 to 12 weeks-old, randomized gender. b) mean ± SEM. c) $CD3^{+}CD19^{-}$. D) $CD19^{+}CD3^{-}$. E) $CD19^{+}CD93^{+}CD1d^{low}$. F) $CD19^{+}CD93^{+}CD1d^{low}IgM^{high}IgD^{low}$. G) $CD19^{+}CD93^{+}CD1d^{low}IgM^{low}IgD^{high}$. H) $CD19^{+}CD93^{-}CD1d^{-}$. i) $CD19^{+}CD93^{-}CD1d^{high}$. J) pooled inguinal, axillary and brachial lymph nodes.

Supplementary Table 4 (related to Figures 7J-K and 8). Data collection and refinement statistics.

	**BAFF-Belimumab Fab**
**Data collection**	
Space group	$P2_1$
Cell dimensions	
a, *b*, *c* (Å)	135.69, 135.50, 138.18
a, b, g (°)	90.0, 91.9, 90.0
Resolution (Å)	2.90 (3.15-2.90) [a]
R_{sym} or R_{merge}	11.9 (57.6)
I / s*I*	11.24 (3.05)
Completeness (%)	98.7 (95.4)
Redundancy	3.36 (3.21)
**Refinement**	
Resolution (Å)	138.11-2.90
No. reflections	108711(654)[b]
R_{work} / R_{free}	0.193 / 0.23.2
No. atoms	
Protein	25957
Water	273
B-factors	
Protein	58.31
Water	34.17
R.m.s. deviations	
Bond lengths (Å)	0.010
Bond angles (°)	1.540

[a] Values in parentheses are for highest-resolution shell.
[b] Randomly selected test set 0.6% of data

Supplementary Table 5. Plasmids used in this study.

**Plasmid**	**Designation**	**Protein encoded**	**Vector**
ps336	FLAG-hBAFF	HA signal-FLAG-GPGQVQLQ-hBAFF (aa 137-285)	PCR3
ps657	FLAG-mBAFF	HA signal-FLAG-GPGQVQLQVD-mBAFF (aa 127-309)	PCR3
ps739	hBCMA-Fc	Ig signal-EVKLVPRGS-hBCMA (aa 2-54)-VD-hIgG1 (aa 245-470)	PCR3
ps837	mBCMA-Fc	Ig signal-DVT-mBCMA (aa 1-46)-VD-hIgG1 (aa 245-470)	PCR3
ps1111	mTACI-Fc	HA signal-LE-mTACI (aa 2-78)-AAAVD-hIgG1 (aa 245-470)	PCR3
ps1377	pMSCS-puro	Modified pMSCV-puro (Clontech) with HindIII-BglII-EcoRI-NotI-XhoI-HpaI-ApaI sites	ps1377
ps1196	Fc-PS-hBAFF	HA signal LD h Fc (h IgG1 aa245-470) RSPQPQPKPQPKPEPEGS-PreSci-GSL h BAFF (aa136-285)	PCR3
ps1910	mBAFF	mBAFF (aa 1-309)	PCR3
ps2297	mBAFFR-Fc	HA signal-LD-mBAFFR (aa 2-70)-VD- hIgG1 (aa 245-470)	PCR3
ps2308	hBAFFR:Fas	HA signal-LE-hBAFFR (aa 2-71)-EFGSVD-hFas (aa 169-355)	ps1377
ps2355	mBAFF H242A	mBAFF (aa 1-309, H242A)	PCR3
ps2394	hBAFF H218A	hBAFF (aa 1-285, H218A)	PCR3
ps2565	His-hBAFF	MRGSHHHHHHGS-h BAFF (aa134-285)	PCR3
ps2566	His-hBAFF H218A	MRGSHHHHHHGS-h BAFF H218A (aa134-285)	PCR3
ps2583	mBAFF E247K	mBAFF (aa 1-309, E247K)	PCR3
ps2784	hBAFF	hBAFF (aa 1-285)	PCR3
ps2825	Fc-hBAFF	HA signal LD h Fc (h IgG1 aa245-470) RSPQPQPKPQPKPEPEGSL h BAFF (aa136-285)	PCR3
ps2846	mBAFF E247K ki	Vector for generation of BAFF E247K knock-in mice	pBluescript
ps2859	mBAFF 5'probe	Vector containing the 5' Southern blot probe (of Suppl Fig. 2) as HindIII/BamHI fragment	pBluescript
ps3195	FLAG-hBAFF H218A	HA signal-FLAG-GPGQVQLQ-hBAFF (aa 137-285, H218A)	PCR3
ps3196	hBAFF E223K	hBAFF (aa 1-285, E223K)	PCR3
ps3198	FLAG-hBAFF E223K	HA signal-FLAG-GPGQVQLQ-hBAFF (aa 137-285, E223K)	PCR3
ps3199	FLAG-mBAFF E247K	HA signal-FLAG-GPGQVQLQVD-mBAFF (aa 127-309, E247K)	PCR3
ps3201	FLAG-mBAFF H242A	HA signal-FLAG-GPGQVQLQVD-mBAFF (aa 127-309, H242A)	PCR3
ps3286	hTACI	hTACI full (aa 1-293)	ps1377
ps3344	psPAX2	Addgene #12260	ps3344
ps3345	pCMV-VSV-g	Addgene #8454	ps3345
ps3391	Lenticrispr v2	Addgene #52961	ps3391
ps3417	gRNA hBAFFR for CRISP/Cas9	5'CACCG**GGCCGAGTGCTTCGACCTGC**-3' and 5'-TTTG**CGTCCAGCTTCGTGAG CCGG**G-3' annealed and cloned into BsmB1 sites of ps3391	ps3391
ps3784	Fc-PS-hBAFF H218A	HA signal LD h Fc (h IgG1 aa245-470) RSPQPQPKPQPKPEPEGS-PreSci-GSL h BAFF (aa136-285, H218A)	PCR3

FLAG = DYKDDDDK HA signal=MAIIYLILLFTAVRG Ig signal=MNFGFSLIFLVLVLKG PreSci= LEVLFGGP

Supplementary reference

1. Holler N, *et al.* Two adjacent trimeric Fas ligands are required for Fas signaling and formation of a death-inducing signaling complex. *Mol Cell Biol* **23**, 1428-1440 (2003).
